# Purification, Characterization, and Structural Studies of a Sulfatase from *Pedobacter yulinensis*

**DOI:** 10.3390/molecules27010087

**Published:** 2021-12-24

**Authors:** Caleb R. Schlachter, Andrea O’Malley, Linda L. Grimes, John J. Tomashek, Maksymilian Chruszcz, L. Andrew Lee

**Affiliations:** 1 Integrated Micro-Chromatography Systems, 110 Centrum Drive, Irmo, SC 29063, USA; caleb.schlachter@imcstips.com (C.R.S.); linda.grimes@imcstips.com (L.L.G.); john.tomashek@outlook.com (J.J.T.); 2 Department of Chemistry and Biochemistry, University of South Carolina, Columbia, SC 29208, USA; aomalley@email.sc.edu

**Keywords:** sulfatase, formylglycine, crystallography, 4-methylumbelliferyl sulfate, hydrolase, *Pedobacter yulinensis*, arylsulfatase

## Abstract

Sulfatases are ubiquitous enzymes that hydrolyze sulfate from sulfated organic substrates such as carbohydrates, steroids, and flavones. These enzymes can be exploited in the field of biotechnology to analyze sulfated metabolites in humans, such as steroids and drugs of abuse. Because genomic data far outstrip biochemical characterization, the analysis of sulfatases from published sequences can lead to the discovery of new and unique activities advantageous for biotechnological applications. We expressed and characterized a putative sulfatase (PyuS) from the bacterium *Pedobacter yulinensis*. PyuS contains the (C/S)XPXR sulfatase motif, where the Cys or Ser is post-translationally converted into a formylglycine residue (FGly). His-tagged PyuS was co-expressed in *Escherichia coli* with a formylglycine-generating enzyme (FGE) from *Mycobacterium tuberculosis* and purified. We obtained several crystal structures of PyuS, and the FGly modification was detected at the active site. The enzyme has sulfatase activity on aromatic sulfated substrates as well as phosphatase activity on some aromatic phosphates; however, PyuS did not have detectable activity on 17α-estradiol sulfate, cortisol 21-sulfate, or boldenone sulfate.

## 1. Introduction

Sulfatases are enzymes found in all biological domains. These enzymes are involved in diverse cellular functions including cell signaling [1], pathogenesis [2], hormone regulation [3], steroid metabolism [4,5] and glycosphingolipid metabolism [6]. Sulfatase nomenclature stems from the type of substrates they are capable of hydrolyzing, e.g., arylsulfatases (small aromatic molecules, EC 3.1.6.1), steryl-sulfatases (steroids; EC 3.1.6.2), glycosulfatases (glucose sulfates, EC 3.1.6.3), N-acetylgalactosamine-6-sulfatases (chondroitin or keratan sulfate, EC 3.1.6.4) and choline sulfatases (choline, EC 3.1.6.6). Arylsulfatases are of medical interest as these sulfatases may be involved in human diseases such as metachromatic leukodystrophy (MLD), Maroteaux–Lamy syndrome (MPSVI), and X-linked Ichthyosis (XLI), all of which are types of multiple sulfatase deficiency in humans (MSD) [7,8]. In addition, arylsulfatases have industrial applications for analyzing steroid sulfates in human urine by mass spectrometry [4,5]. General substrates for sulfatases are compounds or small molecules containing sulfate esters (ROSO_3_^−^) or sulfamates (RN(H)SO_3_^−^) [9].

Barbeyron et al. [9] suggested that the nomenclature of sulfatase enzyme families in the UniProt database [10] reflects the enzyme function. Under such classification, there are four sulfatase families (S1–S4). The largest family of sulfatases is S1, containing sulfatases that remove sulfate ester groups by a hydrolytic mechanism with the metal co-factor calcium [9]. In addition, S1 family sulfatases are unique compared to the other sulfatase families because they contain a modified active site of cysteine or serine that is post-translationally oxidized to a (2S)-2-amino-3-oxopropanoic acid—also referred to as Cα-formylglycine (FGly) [9,11,12]. The consensus amino acid sequence directing the post-translational modification is (C/S)XPXR [12,13,14]. This modification is performed by either an anaerobic sulfatase-maturing enzyme (anSME) or aerobic formylglycine-generating enzyme (FGE) [13,15].

We sought to express and characterize a putative sulfatase from *Pedobacter yulinensis* (Accession No. WP_107215127), a Gram-negative, orange bacterium isolated from sandy soil in the district of Yulin, Shaanxi province, China [16]. The *P. yulinensis* sulfatase, or PyuS, contains the conserved CXPXR sulfatase motif as well as residues for binding the calcium co-factor. PyuS has activity not only on the generic sulfatase substrate 4-methylumbelliferyl sulfate, but also on 5-bromo-4-chloro-3-indolyl phosphate and *para*-nitrophenyl phosphate. PyuS was not able to hydrolyze any of the tested steroid sulfates. Furthermore, we were able to obtain three high-resolution crystal structures of PyuS. The research presented herein describes a previously uncharacterized protein from *P. yulinensis* that has both sulfatase and phosphatase activities, thus providing further insight into the evolutionary and divergent paths that certain enzymes can take over time.

## 2. Results

### 2.1. PyuS Contains the Signature CXPXR Sulfatase Motif

**Figure 1:**
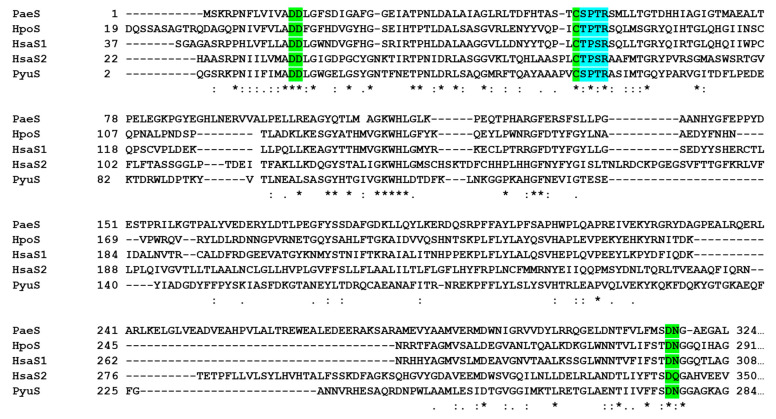
Amino acid sequence alignment of sulfatases from *P. aeruginosa* (PaeS), *H. pomatia* (HpoS), *H. sapiens* (HsaS1 and HsaS2), and *P. yulinensis* (PyuS). Residues involved in binding calcium are highlighted in green, and the conserved CXPXR sulfatase motif (Cys to FGly post-translational modification) is highlighted in teal. HpoS residues for metal binding are based on homology as there is no crystal structure available for this protein. N-terminal signal peptides or putative signal peptides were removed from the alignment where applicable. * Indicates completely conserved residues.

The amino acid sequence of PyuS was chosen using the protein Basic Local Alignment Search Tool (BLASTp, [17]) and searching with the sequences of better characterized sulfatases such as those from *Pseudomonas aeruginosa*, *Helix pomatia*, and *Homo sapiens* (Figure 1). Based on the sequence alignment, PyuS contains the conserved CXPXR sulfatase motif. Furthermore, PyuS also contains conserved active site residues that are involved in coordinating the calcium ion which is necessary for sulfate ester hydrolysis (Figure 1). These residues are Asp15, Asp16, Asp276, Asn277, and FGly55 (modified Cys) in PyuS.

### 2.2. Production and Purification of Recombinant PyuS

The gene for PyuS (Accession No. WP_107215127) was synthesized with a C-terminal His_6_-tag (residues GSHHHHHH) in a cloning vector pUC57 from BioBasic (Toronto, Canada) and codon-optimized for *Escherichia coli* expression. The gene was excised with the restriction enzyme SapI and ligated into the ATUM (Newark, CA, USA) expression vector pD451-SR, which has an inducible T7 promoter. No soluble PyuS was obtained with this initial construct (data not shown). Using the SignalP server [18], it was determined that the first 24 amino acids of PyuS have a 98% possibility of being a signal peptide for a secretory pathway involving the Sec translocon and signal peptidase I (SPI) [19]. The fragment coding for this putative signal peptide was removed from the DNA sequence for further PyuS expression and characterization experiments. In addition, to ensure that the cysteine conversion to formylglycine modification occurred to completion, we co-expressed an FGE from *Mycobacterium tuberculosis* [15,20]. The FGE was codon optimized for *E. coli* expression, and both FGE and PyuS were cloned into an in-house polycistronic plasmid expression vector. In this plasmid, both genes are under the control of an isopropyl *β*-D-1-thiogalactopyranoside (IPTG)-inducible T7 promoter.

PyuS co-expressed with FGE was purified by nickel immobilized metal affinity chromatography (IMAC) and size-exclusion chromatography (SEC) with Tris-based buffers. The yield of recombinant PyuS post-purification was 5 mg per 700 mL of Luria broth (LB) culture. The molecular weight (MW) of recombinant PyuS (without N-terminal signal peptide) is 51.3 kDa (459 residues). Protein concentration was measured by absorbance at 280 nm and using a theoretical molar extinction coefficient at 77,810 M^−^^1^ cm^−^^1^ [21]. Recombinant PyuS has a calculated isoelectric point (pI) of 8.24. For the amino acid sequence numbering convention, we did not include the numbering for the putative signal peptide, i.e., the 25th residue of native PyuS (glutamine) starts as residue number 2 for our recombinant protein (Figure 1). Purified recombinant PyuS (with intact C-terminal His-tag) was used for further characterization.

### 2.3. PyuS Crystal Structure

**Figure 2:**
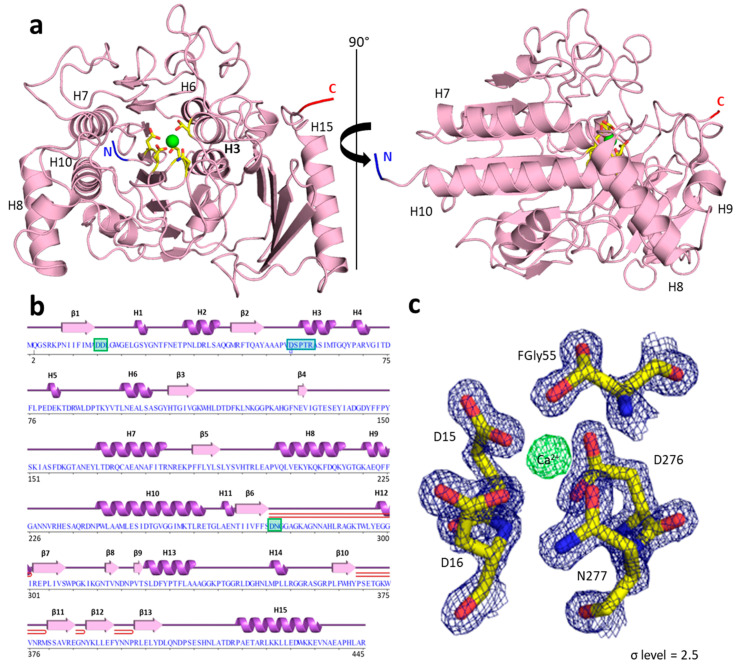
(**a**) Crystal structure of PyuS calcium (green) bound in the active site (PDB code: 7STT). Residues coordinating the calcium ion are shown in yellow. The N- and C-termini are colored in blue and red, respectively. The formylglycine residue is located in helix 3 (**H3**). (**b**) Amino acid sequence and corresponding secondary structural elements of PyuS with labeled α-helices (H) and β-strands (β). The β-hairpins are shown in red. Metal-coordinating residues are highlighted in green. The sulfatase FG motif is highlighted in cyan, and FGly is annotated with a lowercase “d”. (**c**) Electron density map (contoured at 2.5σ) of active site residues coordinating calcium (D15, D16, FGly55, D276, and N277). In the density, the FGly residue can clearly be seen as a hemi-acetal side chain rather than a 2-amino-3-oxopropanoic acid.

Our structural studies show that, despite PyuS having less than 21% sequence identity with the sulfatase sequences in Figure 1, the tertiary structure among them is highly homologous. We were able to obtain three high-resolution crystal structures of recombinant PyuS, most of which were obtained under conditions containing carboxylic acid(s) and had ligand(s) bound to the protein (PDB codes: 7STT, 7STU, 7STV). Figure 2a displays a 1.60 Å crystal structure of recombinant PyuS with calcium bound in the active site (PDB code: 7STT).

Overall, the size of recombinant PyuS is approximately 60 *×* 61 *×* 63 Å (as measured in COOT [22]) and has a calculated solvent-exposed surface area of approximately 17,800 Å^2^ [23]. The protein crystallized in the P3_2_21 space group with one protein chain per asymmetric unit for all structures. The PyuS protein chain has an α/β sandwich fold comprised of 15 α-helices and 13 β-strands that form two anti-parallel β-sheets (Figure 2b). Helix 3 (Figure 2a) contains the CXPXR sulfatase motif and was found in the hydrophobic core of the protein. It is flanked by a β-sheet 1 (β-strands 1–9), which was also found in the protein core, and β-sheet 2 (β-strands 10–13).

The crystal structure of PyuS shows that the active-site cysteine is converted to formyl-glycine. However, with no sulfate substrate bound in the active site, the expected 2-amino-3-oxopropanoic has been oxidized to a hemi-acetal functional group (Figure 2c). This is not an uncommon occurrence among crystal structures of sulfatases with this type of post-translational modification (see PDB codes: 1HDH, 4FDJ, 6G60, 1E33). The aldehyde function of the formylglycine may be stabilized as a hemi-acetal functional group, as evidenced by experiments with sodium borohydride and guanidine-denatured sulfatases [24]. In addition, the analysis of the electron density maps, B-factors, and geometry of the metal binding site allowed us to identify Ca^2+^ as being present in the active site (Figure 2c). Based on our analysis, we assigned a metal occupancy of 60%. The metal binding site was validated with CheckMyMetal server [25]. The calcium ion was further verified by inductively coupled plasma-mass spectrometry (ICP-MS). In a purified sample of PyuS not supplemented with calcium, 165 ppb (µg/L) Ca^2+^ was detected, which corresponds to approximately two Ca^2+^ cations per PyuS molecule. The concentration of magnesium in the sample was also determined, and the protein sample contained only 7.3 ppb of Mg^2+^. These results clearly indicate that the recombinant PyuS sample contains calcium bound in the active site.

### 2.4. PyuS Oligomerization

**Figure 3:**
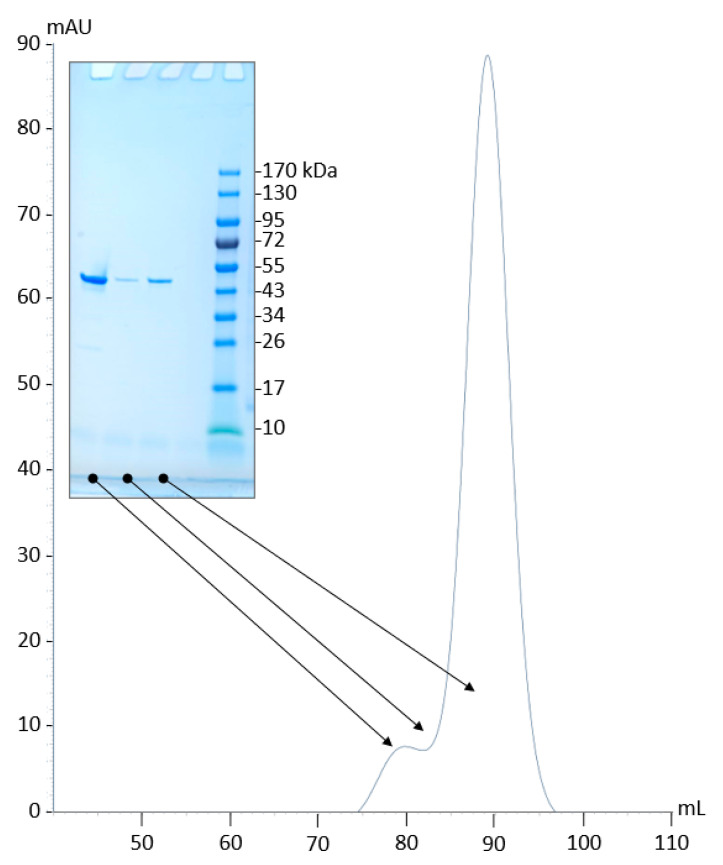
SDS-PAGE gel and size exclusion chromatography purification of recombinant PyuS. For SEC, the absorbance was monitored at 280 nm. The arrows indicate the elution fraction analyzed by SDS-PAGE and the MW of PyuS is 51.3 kDa.

The PDBePISA [23] analysis of PyuS crystal structures revealed that PyuS forms homodimers. However, results from size exclusion chromatography (SEC) suggest that only a small population of PyuS dimers may exist in solution. The purification of recombinant PyuS by SEC in Tris-buffered saline (TBS) gave two elution peaks (Figure 3). Proteins from both elution peaks were analyzed by sodium dodecyl sulfate polyacrylamide gel electrophoresis (SDS-PAGE) under reducing conditions (Figure 3), and both peaks show a protein band at approximately 50 kDa. SEC MW standards were used to calculate the MW of both peaks (Supplementary Figure S1); as expected, the MW of the first elution peak is approximately twice as large as the second elution peak, suggesting that the peaks represent dimers and monomers, respectively.

**Figure 4:**
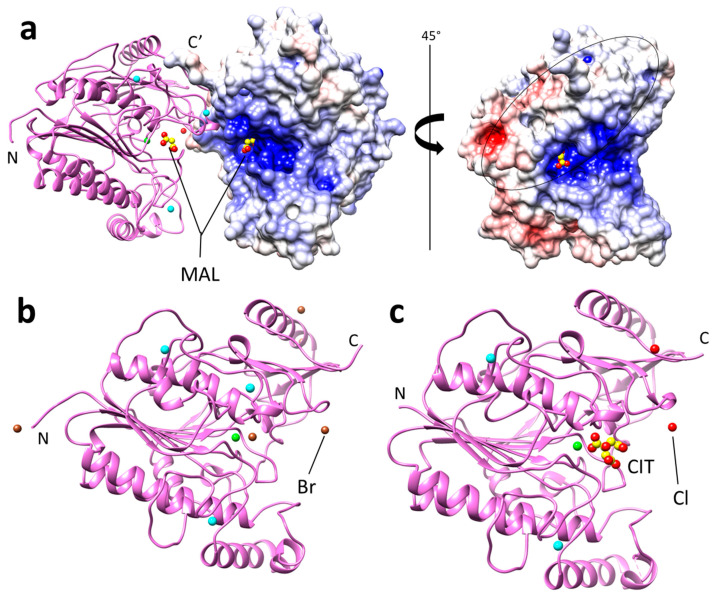
(**a**) Predicted homodimer of PyuS (PDB code: 7STT) with calcium (green) and malonate (MAL) bound in the active site, as well as sodium (teal) and chlorine (red) ions bound on the protein surface. The dimer interface is mainly formed through electrostatic interactions (black oval). One monomer is shown in cartoon representation (pink) while the other is shown in a surface representation of the electrostatic map (red indicates negative charge, white indicates neutral charge, and blue indicates positive charge). The N indicates the N-terminal of monomer 1, and C’ indicates the C-terminal of monomer 2. (**b**) PyuS crystal structure 7STU with sodium (teal), calcium (green), and bromine (brown) ions bound. (**c**) PyuS crystal structure 7STV with citrate (CIT) and sodium (teal), calcium (green), and chlorine (red) ions bound.

In the 7STT PyuS crystal structure, the interface area of a PyuS homodimer is calculated to be 1023 Å^2^. This interface area is above the cut-off value of 856 Å^2^ which was established by Ponstingl et al. [26] for differentiating between monomeric and homodimeric proteins in the crystalline state. However, the interface area calculated between monomers in 7STU (bromine) and 7STV (citrate) crystal structures were 876 Å^2^ and 793 Å^2^, respectively, though the differences in interface areas could be attributed to the crystallization conditions. A PyuS homodimer structure generated by PDBePISA [23] is shown in Figure 4a for PDB code: 7STT. The interface mainly consists of intercalated, charged residues from Helix 5 (residues 77–85), β-hairpin 2 (residues 369–379), and the C-terminal end (residues 439–445) of each monomer in the dimer. In addition, the C-terminal is more disordered in the 7STU and 7STV structures compared to 7STT, and the C-terminal His-tag is not visible in the electron density for any of the structures. For 7STT (Figure 4a), there is also a Na^+^ binding site involving residues Asp80 and Thr83. The 7STU (Figure 4b) and 7STV (Figure 4c) structures contain Br^-^/Cl^-^ binding sites (residues Asn377 and Arg 378), respectively, between their monomer interfaces.

### 2.5. PyuS Activity on Sulfatase and Phosphatase Substrates

#### 2.5.1. PyuS Activity on Aromatic Sulfate Esters and Steroid Sulfates

**Figure 5:**
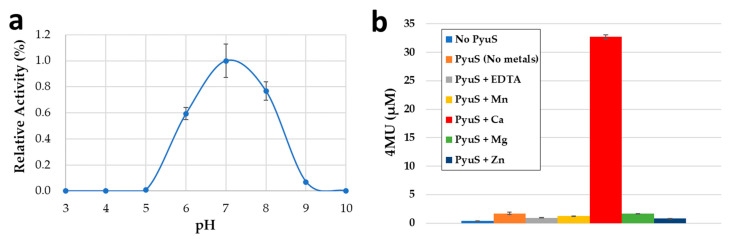
(**a**) The relative pH optima of PyuS as determined with sulfatase substrate 4MUS. (**b**) Activity of PyuS on 4MUS in the presence of the different metals. No PyuS, PyuS (No metals), and EDTA were used as controls. Error bars represent biological replicates.

Initially, the small, aromatic, fluorescent substrate 4-methylumbelliferyl sulfate (4MUS) was used to determine the optimal pH range for recombinant PyuS (Figure 5a). PyuS has the highest activity around neutral pH in the range of 6.5–7.5. In addition, PyuS was supplemented with manganese, calcium, magnesium, or zinc, and the activity at pH 6.5 was determined with 4MUS (Figure 5b).

Based on the metal supplement assay with substrate 4MUS, calcium is the preferred co-factor for PyuS, as expected based on data from the crystal structures (Figure 2c). The PyuS sample (no metals) in Figure 5b contains 4MUS substrate but does not contain any supplemented co-factor; comparing only PyuS to PyuS + Ca^2+^, it is apparent that adding additional calcium to the reaction will increase the activity of PyuS and provides evidence that calcium is the preferred co-factor for the catalysis of sulfate esters. In addition, the metal chelator ethylenediaminetetraacetic acid (EDTA) was used as a negative control for the 4MUS reactions (Figure 5b), and as expected, PyuS had lower activity in the presence of the EDTA when compared to the PyuS (no metals) sample.

**Figure 6:**
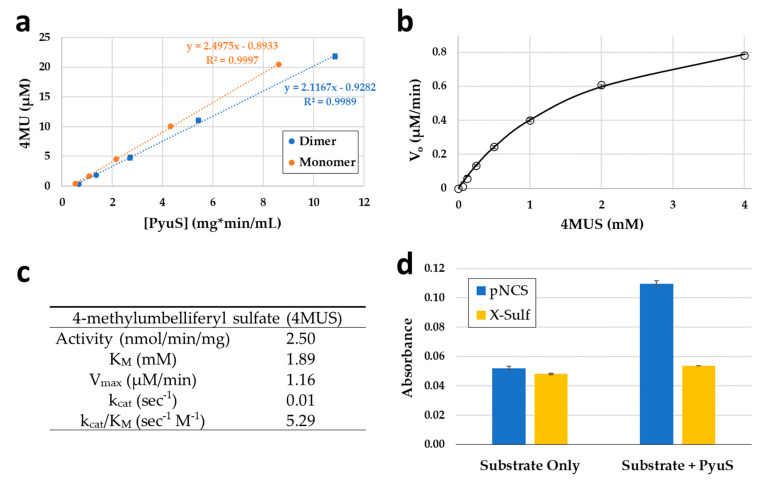
(**a**) Activity curves of dimeric and monomeric recombinant PyuS on substrate 4MUS. (**b**) Enzyme kinetics curve for 4MUS fit with the Michaelis–Menten equation. (**c**) PyuS kinetic parameters on 4MUS substrate. (**d**) Recombinant PyuS activity on arylsulfatase substrates pNCS and X-Sulf at pH 6.5. The pNCS and X-Sulf products were measured at 515 nm and 650 nm, respectively. Assays were performed with biological replicates.

The dimeric and monomeric elution samples from SEC (Figure 3) were concentrated separately, and their activities on 4MUS were compared at pH 6.5 (Figure 6a). The dimeric PyuS has an activity of 2.12 nmol/min/mg on 4MUS, whereas the monomeric PyuS has an activity of 2.50 nmol/min/mg. These results indicate that the oligomerization state of PyuS does not have a substantial impact on 4MUS activity. The activity of the monomer on 4MUS was investigated further with Michaelis–Menten kinetics (Figure 6b,c). Because the enzyme appears to exist as both dimer and monomer, kinetic data were modeled by using both the Michaelis–Menten equation and the Hill equation. The Michaelis constant (K_m_) was calculated to be 1.89 mM, the velocity max (V_max_) at 1.16 µM/min, turnover number (k_cat_) at 0.59 min^−^^1^, and a Hill coefficient of approximately 1.0 was determined, suggesting that there is no obvious cooperativity between PyuS active sites.

PyuS activity with two other general arylsulfatase substrates, *para*-nitrocatechol sulfate (pNCS) and 5-bromo-4-chloro-3-indoxyl sulfate (X-Sulf), was also determined (Figure 6d). Compared to 4MUS hydrolysis, the activity of PyuS on pNCS is remarkably slow; it should be noted that the 4MUS assay was incubated for 21 h (Figure 6a), whereas the pNCS assay (Figure 6d) was incubated for over 48 h, resulting in low product formation (see Materials and Methods for more details). PyuS did not have any significantly apparent activity on X-Sulf (Figure 6d), another substrate which has been used for arylsulfatase screening [27].

Some arylsulfatases, such as PaeS from *P. aeruginosa*, are capable of hydrolyzing steroid sulfates [4,5]. The activity of PyuS on the steroid sulfates cortisol 21-sulfate (CS), 17α-estradiol sulfate (αES), and boldenone sulfate (BS) was measured by high-pressure liquid chromatography (HPLC). However, PyuS was not able to hydrolyze any of the tested steroid substrates (data not shown).

#### 2.5.2. PyuS Activity on Phosphatase Substrates

**Figure 7:**
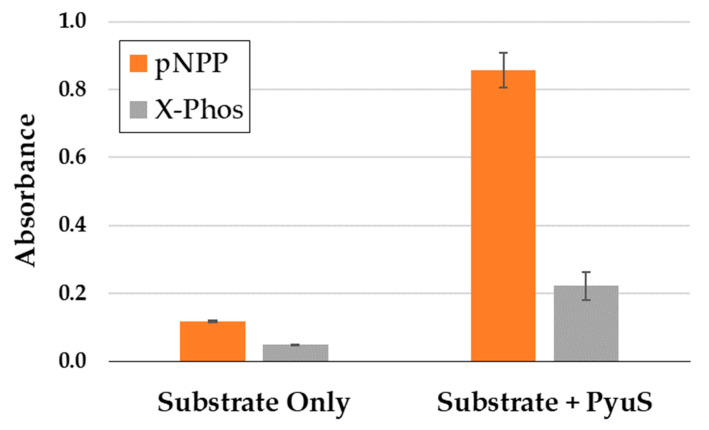
Activity of PyuS on phosphatase substrates pNPP and X-Phos. Products of pNPP and X-Phos were measured at 405 nm and 650 nm, respectively. Assays were performed with biological replicates.

Due to the evolutionary relationship of sulfatase and alkaline phosphatase enzyme families [28] and the promiscuity of both activities, the phosphatase substrates *para*-nitrophenyl phosphate (pNPP) and 5-bromo-4-chloro-3-indoxyl phosphate (X-Phos) were incubated with PyuS to monitor phosphatase activity. Interestingly, PyuS is able to hydrolyze both pNPP and X-Phos (Figure 7).

### 2.6. PyuS Stability

**Figure 8:**
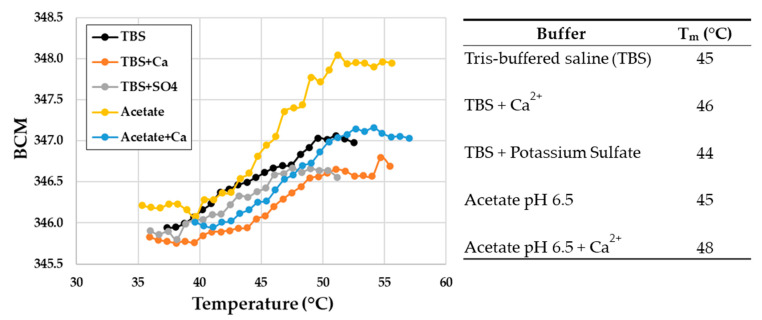
Melting curves (T_m_) for recombinant PyuS in various buffers. Samples were biological replicates.

The melting temperature (T_m_) of recombinant PyuS was determined by measuring the change in intrinsic tryptophan fluorescence emission wavelength due to changes in the polarity of the surrounding environment, and this value was recorded as the barycentric mean (BCM) (Figure 8). Stability was measured in TBS, TBS with calcium, TBS with potassium sulfate, sodium acetate pH 6.5, and sodium acetate with calcium. The condition of sodium acetate pH 6.5 supplemented with calcium resulted in the highest T_m_ of approximately 48 °C, whereas all other conditions had a T_m_ of approximately 45 °C. Sodium acetate was tested because it was the buffer of choice for hydrolysis assays.

## 3. Discussion

We showed that the putative sulfatase PyuS from *P. yulinensis* contains the sulfatase motif CXPXR and that the cysteine is post-translationally converted into a formylglycine when co-expressed with an FGE in *E. coli*. However, in the electron density of the crystal structures, the formylglycine is observed as a hemi-acetal group, and it is unclear whether this is the naturally preferred resting state of the residue or just an artifact of purification or crystallization. PyuS is most active near neutral pH 7 on substrate 4MUS, uses calcium as a cofactor, and hydrolyzes the sulfatase substrates 4MUS and pNCS, as well as phosphatase substrates pNPP and X-Phos. However, it has no detectable activity on steroid sulfates αES, CS, or BS. Calcium was the co-factor of choice for 4MUS hydrolysis, but it is possible that PyuS may utilize a different co-factor, such as magnesium, for the hydrolysis of other substrates, and this concept would need to be explored further.

Based on size-exclusion chromatography, approximately 10% of soluble PyuS will form homodimers (Figure 3). Dimers are also seen in the crystal structures, and PDBePISA [23] shows that the interface area between monomers in the crystal structure exceeds the threshold to distinguish between real dimers and experimental artifacts. Exploring the interface area further reveals that mostly electrostatic interactions permit dimerization, and there are no inter- or intramolecular disulfide bonds (other than the Cys in CXPXR motif, PyuS only has one cysteine residue which is not solvent exposed). In all of the PyuS crystal structures (Figure 4), there is at least one type of ion (sodium, bromine, or chloride) present in the putative dimer interface, and these ions may help facilitate dimer formation.

It should be reiterated that the C-terminal electron density was disordered for all structures, where 7STU and 7STV C-termini had more disorder than that in 7STT. The C-terminal ends in the putative dimer (Figure 4a) are only approximately 20 Å apart as measured from the Cα’s of Arg445 from each monomer. At this proximity, it is plausible that the His-tag from each monomer could interact with one another [29,30], and the dimers we observe during purification and from crystallization could be a consequence of this interaction. However, it should also be considered that the His-tag could be preventing oligomerization, and if the His-tag was moved to the N-terminal or if a different purification tag was used altogether, it is possible that PyuS oligomerization would be more prominent.

Although a low percentage of soluble PyuS is in an oligomeric state, we cannot rule out the possibility that this may change in the presence of substrate or by increasing the concentration of PyuS. The activity of the dimeric and monomeric PyuS (Figure 4a) was compared on 4MUS, and there was a minimal difference in activity observed (Figure 6a). In the homodimer, the active sites are approximately 33 Å apart as measured between active site calcium ions in PyMOL [31]. The cooperativity could be further investigated by modifying residues in Helix 5 (residues 77–85) and β-hairpin 2 (residues 369–379, Figure 2b) as these structural elements are in close proximity to the active site of the opposing monomer in the homodimer.

Because there are generally low sequence identities among sulfatases, we used several homologous protein structure-search programs such as PDBeFold [32,33], Dali [34], PDBsum [35], CATH [36,37], and PDBeMotif [38] to find homologous structures to PyuS (Table 1).

As expected, sulfatases were the most prominent homologous proteins found when searching with the PyuS structure, but there were a few alkaline phosphatase structures in the list as well which had lower homology than the sulfatases (Table 1). Interestingly, the sulfatases with high homology to PyuS had variable functions which include N-acetylgalactosamine-4-sulfatases, N-acetylglucosamine-6-sulfatases, arylsulfatases, sterylsulfatases, and Iota-carrageenan sulfatases (Table 1). The sulfatase structures in Table 1 were aligned with PyuS in PyMOL [31], and the root mean square deviation (RMSD) was generally close to 1.0 Å or better when aligning the structures by alpha carbons (Cα).

The active site of PyuS contains His113, His195, and Lys293 which are completely conserved among the sulfatases listed in Table 1. In addition, the PyuS active site contains Phe76 that is not conserved among sulfatases with known crystal structures in the PDB. For example, Phe76 is replaced by tyrosine in the *H. sapiens* N-acetylgalactosamine-6-sulfatase (PDB code: 4FDJ) or tryptophan in the *Bacteroides fragilis* sulfatase (PDB code: 6USS). Our structural studies indicate that negatively charged entities such as malonate, bromine, and citrate are favored ligands for binding to the PyuS active site, and these ligands are in the vicinity of active site residues His195 and Lys293. This is not surprising as they mimic sulfate or the phosphate moieties of sulfatase or phosphatase substrates, respectively, and the active site of PyuS has an overall positive charge (Figure 4a).

A Cα alignment of all the sulfatase structures in Table 1 shows that the sulfatase active site is comprised of several unstructured loops, similarly to PyuS (Figure 2a), and it is not far-fetched to believe these loops influence substrate specificity and the pH optima of the enzymes. Based on our kinetic studies, the sequence alignments, and structure alignments, it is plausible that PyuS is actually an N-acetylgalactosamine-4-sulfatase or N-aceteylglucosamine-6-sulfatase instead of an arylsulfatase. If that is not true, then PyuS may be involved in the metabolic pathways of *P. yulinensis,* and scavenging sulfate [40] or phosphate-containing [41] compounds may be its primary role.

## 4. Materials and Methods

### 4.1. Gene Synthesis, Protein Expression, and Purification

The amino acid sequence of PyuS (Accession No. WP_107215127) was found by using the protein Basic Local Alignment Search Tool (BLASTp, [17]) with sequences from *P. aeruginosa* (Accession No. WP_003106692), *H. pomatia* (Accession No. AF109924), or *H. sapiens* (Accession No. NP_000037, AAA60596). A codon-optimized nucleic acid sequence for PyuS was synthesized by BioBasic (Toronto, Canada) for *E. coli* expression. The N-terminal region of native PyuS (amino acid residues 2–24) was predicted to be a signal peptide by the SignalP server [18], and these residues were removed from the DNA sequence for expression. A C-terminal His6-tag (residues GSHHHHHH) was added for IMAC purification, and the final molecular weight of the recombinant PyuS construct was 51,320 Da (459 amino acids). In addition, the FGE gene from *M. tuberculosis* was ordered from BioBasic (Toronto, Canada) for co-expression with PyuS in *E. coli* [15,20]. Both genes were cloned into pCRS195, a proprietary plasmid synthesized (by Life Technologies Corporation, Carlsbad, CA) for use by Integrated Micro-Chromatography Systems (IMCS). Genes were incorporated into pCRS195 with Bsu36I, BamHI, AvrII, and MfeI restriction sites; restriction enzymes were purchased from New England Biolabs (NEB, Ipswich, MA, USA). In pCRS195, genes are expressed under the control of a repressible T7 promoter. The resulting plasmid containing both FGE and PyuS genes was named pCRS268.

*E. coli* T7 expressing cells from NEB (Ipswich, MA, USA) were transformed with pCRS268 and grown on LB-kanamycin (KAN, 50 µg/mL) agar plates. A starter culture was made from a single colony in LB-KAN, and this culture was grown overnight at 37 °C. Starter culture was added to 700 mL of LB-KAN, grown to O.D. 0.8 at 37 °C, and a working concentration of 50 µM copper sulfate was added [42]. The culture was cooled at 16 °C for 10 min and then induced at 16 °C with 0.1 mM isopropyl β-D-1-thiogalactopyranoside (IPTG) for 16 h. Cell pellets were harvested and frozen at −80 °C until further needed.

Cells were lysed with Bacterial Protein Extraction Reagent (B-PER™, ThermoFisher Scientific, Waltham, MA, USA) following the manufacturer’s protocol. Soluble cell lysate was purified with a 1.0 mL nickel-IMAC column attached to an ÄKTA pure protein purification system (Cytiva, Marlborough, MA. USA) at 4 °C. The loading buffer used was 10 mM Tris, 100 mM sodium chloride, 10 mM imidazole, pH 8. The elution buffer was the same but with the addition of 500 mM imidazole. The IMAC column was washed with 5%, 10%, 40%, and 100% of elution buffer, and PyuS was found in both the 10% and 40% elution fractions. Protein was concentrated with an Amicon concentrator (EMD Millipore, Burlington, MA, USA) with a 30 kDa cut-off membrane. Protein was purified further by SEC with a Cytiva HiLoad 16/600 superdex 200 pg column, using 10 mM Tris, 150 mM sodium chloride, pH 8.0 as the running buffer at a flow rate of 1.2 mL/min and 4 °C. Protein purity was analyzed by using pre-cast 4%–20% gradient SDS-PAGE gels from BioRad (Hercules, CA, USA). Protein concentration was measured by determining the absorbance at 280 nm and using the ExPASy [21] predicted extinction coefficient of 77,810 M^−1^ cm^−1^. PyuS has an isoelectric point of 8.24. The recombinant PyuS yield from a 700 mL LB culture was approximately 5 mg.

### 4.2. Metal Analysis

The presence of calcium and magnesium in the protein was evaluated with inductively coupled plasma–mass spectrometry (ICP-MS). Nitric acid was added to PyuS (0.5 mg protein in 500 µL of 10 mM Tris, 150 mM NaCl, pH 8) to a final concentration of 2% HNO_3_ before analysis. A Finnigan ELEMENT XR double focusing magnetic sector field (SF) ICP-MS was used for the analysis with Rh as an internal standard. A 0.2 mL/min Micromist U-series nebulizer (GE, Port Melbourne, Victoria, Australia), quartz torch, and injector (Thermo Fisher Scientific, Waltham, MA, USA) were used for sample introduction. Five-point calibration curves were used in a calibration range from 10 to 600 ppb.

### 4.3. Protein Crystallization, Data Collection, and Refinement

Protein crystals were obtained via the vapor diffusion technique. The 96-well crystallization plates (Hampton Research, Aliso Viejo, CA, USA) were left at room temperature (23 °C) for all experiments. Crystallization drops were set with 1.0 µL of protein solution with protein concentrations varying from 4 to 6 mg/mL in 10 mM Tris, 150 mM NaCl, pH 8.0 buffer. The protein was mixed with 1.0 µL of crystallization solution in the wells. Protein was also incubated on ice for 30 min with sulfatase irreversible inhibitor STX64 (Axon Medchem, Reston, VA, USA) [43,44] with a final inhibitor concentration of 1.0 mM before setting drops. However, the inhibitor did not solubilize well in the protein mixture and precipitated out of solution in the crystallization drops. No crystals were found to contain the STX64 inhibitor.

Protein crystals were grown in wells containing 2.4 M sodium malonate pH 7.0 (PDB code: 7STT), 0.15 M potassium bromide and 30% *w*/*v* polyethylene glycol monomethyl ether 2000 (PDB code: 7STU), or 0.1 M sodium citrate pH 5.5 and 20% *w*/*v* polyethylene glycol 3000 (PDB code: 7STV). Crystals were cryocooled in liquid nitrogen prior to data collection. All X-ray diffraction experiments were performed at the South East Regional Collaborative Access Team beamline 22ID at the Advanced Photon Source (Argonne National Laboratory, Lemont, IL, USA). HKL-2000 was used for data processing [45]. Data collection statistics for the three structures are reported in Table 2.

### 4.4. Structure Determination, Refinement, and Validation

The structures were solved by molecular replacement using MOLREP [46] and HKL-3000 [47]. The search model for 7STT was a predicted structure from SWISS-Model [48,49,50] using a structure of an N-acetylglucosamine-6-sulfatase (PDB code: 6UST) with 38.7% sequence identity to PyuS. The 7STT structure was then used as the search model for the other two structures (7STU and 7STV). The structures were refined with REFMAC [51] and COOT [22]. The TLS Motion Determination server [52] was used to split the single protein chain into fragments in the final stages of refinement for each of the three structures. MOLPROBITY [53] and COOT were used for structure validation. The identities and locations of metals in the structures were validated with the CheckMyMetal (CMM) server [25,54,55]. Table 1 shows the final refinement and validation statistics. The three structures were deposited in the PDB with accession codes 7STT, 7STU, and 7STV.

### 4.5. Protein Modeling and Structure Analysis

The predicted crystal structure of HpoS was generated by using SWISS-Model [48,49,50]. Protein structures were analyzed with PDBePISA [23], PDBeFold [32,33], Dali [34], PDBsum [35], CATH [36,37], and PDBeMotif [38]. Protein crystal structures were modeled in COOT [56], PyMOL [31] or Chimera [57]. Electrostatic maps were generated or modified with APBS [58] or CCP4 package suite [59]. Protein sequences were aligned with the program CLUSTAL W [39].

### 4.6. Hydrolysis Assays with 4MUS, pNCS, and X-Sulf

All chemical structures of substrates can be found in Supplementary Figure S2. Substrates were purchased from Sigma-Aldrich (St. Louis, MO, USA), GoldBiotechnology (St. Louis, MO, USA), or Fisher Scientific (Waltham, MA, USA). All other reagents were purchased from Sigma-Aldrich (St. Louis, MO, USA) or ThermoFisher (Waltham, MA, USA). The pH optimum of PyuS was determined using a triple buffer system of acetic acid, MES, and Tris as described by Ellis and Morrison [60] with 4-methylumbelliferyl sulfate at a working concentration of 1.0 mM, calcium chloride at 1.0 mM, and PyuS at approximately 0.05 mg/mL at room temperature (RT) for 2 h. The metal co-factor assay used a working concentration of 1.0 mM manganese chloride, calcium chloride, magnesium chloride, zinc acetate, or EDTA in 100 mM sodium acetate, pH 6.5, with 0.2 mg/mL PyuS and 1.0 mM 4MUS for 3 h at RT. The 4MUS standard assay was performed with a working concentration of 1.0 mM 4MUS and 0.5 mM calcium chloride in 50 mM sodium acetate, pH 6.5, for 21 h at RT. The *para*-nitrocatechol sulfate (pNCS) and 5-bromo-4-chloro-3-indoxyl sulfate (X-Sulf) assays were performed at 1.0 mM and 0.3 mg/mL concentrations, respectively, in 100 mM sodium acetate pH 6.5, 0.5 mM calcium chloride, and 0.3 mg/mL PyuS with a 60 h incubation at RT. All 4MUS, pNCS, and X-Sulf reactions were stopped with 200 mM glycine, pH 10.4. Excitation and emission wavelengths used for the detection of 4-methylumbelliferone (4MU) were 360 nm and 449 nm, respectively. The absorbance of products from pNCS and X-Sulf hydrolysis were detected at 515 nm and 650 nm, respectively. All assay products were detected in a Spectramax M5 (Molecular Devices, San Jose, CA, USA). Hydrolysis assays were performed in duplicate.

For Michaelis–Menten kinetics, the highest working concentration was 4.0 mM 4MUS. The 4MUS was serial diluted 1:1 in 25 mM Bis-Tris, pH 6.5, and 1.0 mM calcium chloride, to 0.06 mM 4MUS which served as the lowest concentration. The working concentration of PyuS was 0.1 mg/mL, and reactions were 50 µL total and proceeded for up to 1.5 h at room temperature. Reactions were stopped with the addition of 150 µL 200 mM glycine, pH 10.4. All reactions were performed in duplicate except 4.0 mM and 0.06 mM 4MUS, which were single reactions. Kinetic parameters were determined with the Michaelis–Menten or Hill equation and by using the Microsoft Excel Solver add on.

### 4.7. HPLC with Steroid Sulfates

Steroids were purchased from Toronto Research Chemicals (Toronto, ON, Canada) or Cerilliant (Round Rock, TX, USA). For assay setup, 0.5 mg/mL PyuS in 100 mM sodium acetate pH 6.5 and 1.0 mM calcium chloride was mixed with 0.1 mg/mL 17α-estradiol sulfate or 0.01 mg/mL cortisol 21-sulfate or boldenone sulfate at RT for 24 h. Samples were stopped with 41% MeOH, 11% acetonitrile, 10 mM potassium phosphate pH 7.6, and 0.1% formic acid (*v*/*v*). Ultra-performance liquid chromatography was performed on a Thermo-Scientific™ Vanquish™ UHPLC system using a Thermo Scientific™ Accucore™ biphenyl reversed phase 80 Å column (2.1 mm × 50 mm, 2.6 µm). The column was heated to 35 °C and used with a flow rate of 0.5 mL/min. Mobile phase used was HPLC running buffer: 41% methanol, 11% acetonitrile, 10 mM potassium phosphate, pH 7.6. The absorbance was measured at 250 nm for CS and BS, and 280 nm for αES. PyuS had no detectable activity on these steroids. Samples were performed in duplicates.

### 4.8. Hydrolysis Assays with pNPP and X-Phos

The *para*-nitrophenyl phosphate was purchased from Millipore Sigma (St. Louis, MO, USA), and X-Phos was purchased from Fisher Scientific (Waltham, MA, USA). The pNPP and X-Phos were at 1.0 mM and 0.4 mg/mL, respectively, with 100 mM sodium acetate pH 6.5, 0.5 mM calcium chloride, and 0.3 mg/mL PyuS incubated at RT for 60 h. Reactions were stopped with 200 mM glycine, pH 10.4. Products of pNPP and X-Phos were read at wavelengths of 405 nm and 650 nm, respectively, in a Spectramax M5 (Molecular Devices, San Jose, CA, USA). Samples were performed in duplicates.

### 4.9. Melting Temperature of PyuS

The melting temperature for PyuS was measured by using an UNcle instrument from UNchained Labs (Woburn, MA, USA). Protein was used at a working concentration of 0.8 mg/mL, calcium chloride at 1.0 mM, potassium sulfate at 1.0 mM, TBS at 10 mM Tris, 150 mM sodium chloride (pH 8.0), and sodium acetate at 100 mM where applicable. Samples were heated for 180 s at different temperatures in 1.0 °C increments, ranging from 30 °C to 95 °C. The change in intrinsic tryptophan fluorescence emission wavelength was measured at each temperature and recorded as BCM. The melting temperature was calculated from the inflection point of the first derivative. Samples were in duplicates and the standard deviation for all samples was less than zero.

## Figures and Tables

**Table 1:** Protein Data Bank (PDB) sulfatase structures homologous to PyuS. RMSD is the root mean square deviation from Cα alignment; Cα is the number of alpha carbons aligned; and sequence identity refers to primary amino acid sequence alignment. RMSD values were calculated by aligning Cα in PyMOL [31]. Sequence identity was calculated with CLUSTAL W [39]. Signal peptides or putative signal peptides were removed for all alignments.

PDB Code	Protein	RMSD (Å)	Cα Aligned	Sequence Identity to PyuS (%)
1FSU	N-acetylgalactosamine-4-sulfatase	1.0	265	18.4
1HDH	Arylsulfatase	1.7	260	15.3
1P49	Sterylsulfatase	0.9	251	20.0
6USS	Sulfatase (not classified)	0.8	275	24.9
6UST	N-acetylglucosamine-6-sulfatase	0.8	317	29.8
5G2V	N-acetylglucosamine-6-sulfatase	1.7	251	19.8
4FDJ	N-acetylglucosamine-6-sulfatase	0.9	249	23.3
6B0J	Iota-carrageenan sulfatase	0.9	277	23.1

**Table 2:** Data collection and refinement statistics for PyuS crystal structures. Values in parentheses are for the highest resolution shell. RMSD—root mean square deviation.

Structure	PyuS (Malonate)	PyuS (Bromine)	PyuS (Citrate)
PDB accession code	7STT	7STU	7STV
**Data collection**			
Diffraction source	APS, 22ID	APS, 22ID	APS, 22ID
Wavelength (Å)	1.0	1.0	1.0
Space group	P3_2_21	P3_2_21	P3_2_21
a, b, c (Å)	83.6, 83.6, 116.1	83.6, 83.6, 115.8	82.7, 82.7, 115.3
Resolution range (Å)	40.0–1.60 (1.63–1.60)	40.0–2.23 (2.27–2.23)	40.0–2.34 (2.38–2.34)
No. of unique reflections	59,449 (2069)	23,149 (1144)	19,275 (936)
Completeness (%)	95.7 (67.5)	99.2 (99.6)	98.8 (98.7)
Redundancy	10.6 (5.8)	9.1 (6.2)	9.2 (6.7)
<I/σ(I)>	35.3 (2)	15.1 (2)	18.5 (2)
Rmeas	0.079 (0.487)	0.172 (0.858)	0.148 (0.525)
Rp.i.m.	0.024 (0.186)	0.057 (0.339)	0.048 (0.175)
CC1/2	0.996 (0.859)	0.997 (0.738)	0.998 (0.948)
**Refinement**			
Resolution range (Å)	39.37–1.60 (1.63–1.60)	39.22–2.23 (2.29–2.23)	34.23–2.35 (2.41–2.35)
Completeness (%)	95.5 (68.4)	99.2 (99.3)	98.4 (93.3)
No. of reflections, working set	56312	21815	18285
No. of reflections, test set	3033	1205	943
Final Rcryst	0.148 (0.276)	0.174 (0.282)	0.172 (0.286)
Final Rfree	0.178 (0.270)	0.225 (0.319)	0.230 (0.301)
RMSD bonds (Å)	0.014	0.007	0.005
RMSD angles (°)	1.8	1.4	1.3
**Ramachandran plot**			
Allowed regions (%)	100	100	100
Favored regions (%)	97.1	97.1	97.2

## Data Availability

All crystal structures are available from the Protein Data Bank (PDB) with accession codes 7STT, 7STU, and 7STV.

## Supplementary Materials

The following are available online, Figure S1: SEC purification of PyuS. Figure S2: Chemical structure of substrates characterized with PyuS.

## Sample Availability

Raw diffraction images are available from the authors upon request.
