# Supplementary material for: Purification, Characterization, and Structural Studies of a Sulfatase from Pedobacter yulinensis
Source: Molecules. 2021 Dec 24;27(1):87. doi: 10.3390/molecules27010087 (PMC8746622; doi:10.3390/molecules27010087)
Supplement: Supplementary file 1 [file molecules-27-00087-s001.zip › molecules-1511897-supplementary.pdf]

Purification, Characterization, and Structural Studies of a Sulfatase from *Pedobacter Yulinensis*

Caleb R. Schlachter[1], Andrea O'Malley[2], Linda L. Grimes[1], John J. Tomashek[1], Maksymilian Chruszcz[2*] and L. Andrew Lee[1*.]

Supplementary Figures

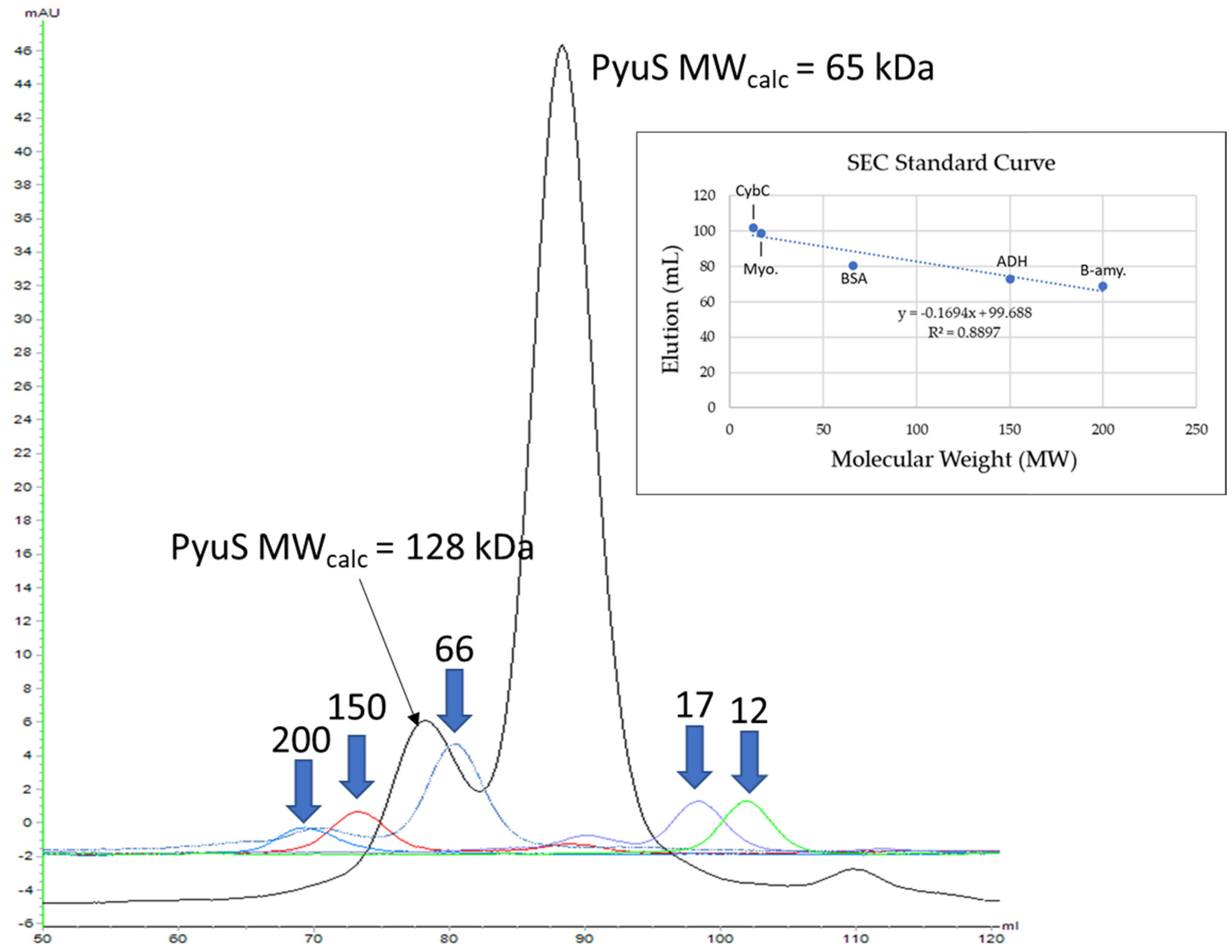

Supplementary Figure S1. Size exclusion chromatography (SEC) standards and PyuS SEC results. Blue arrows show elution standards. Black peak is elution for PyuS. PyuS, according to the standard curve generated here, contained two peaks with MW_{calc} of 128 kDa and 65 kDa, respectively; the MW_{calc} from the amino acid sequence alone is 51.3 kDa. SEC was performed on HiLoad 16/600 superdex 200 pg (from Cytiva) at 1.2 mL/min with 10 mM Tris, 150 mM NaCl, pH 8. Standards were purchased from Cytiva, Sigma-Aldrich, or ThermoFisher. CybC – cytochrome c from bovine heart (12.4 kDa), Myo. – myoglobin (17 kDa), BSA – bovine serum albumin (66 kDa), ADH – alcohol dehydrogenase (150 kDa), B-amy. – beta-amylase (200 kDa).

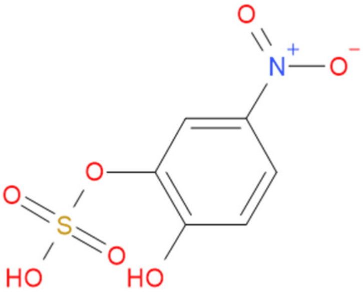

(1)

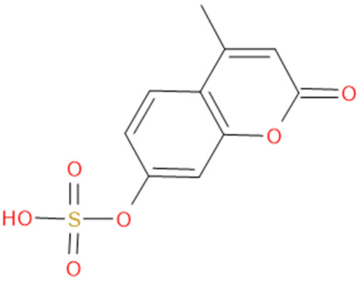

(2)

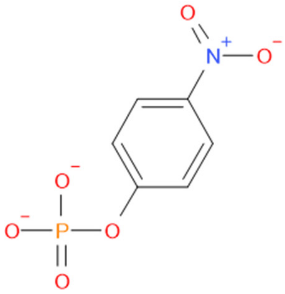

(3)

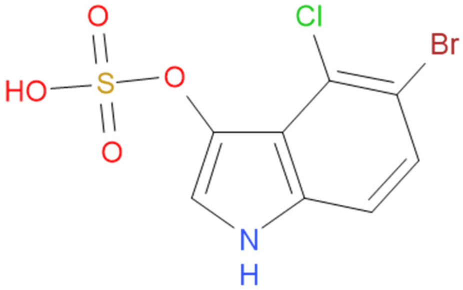

(4)

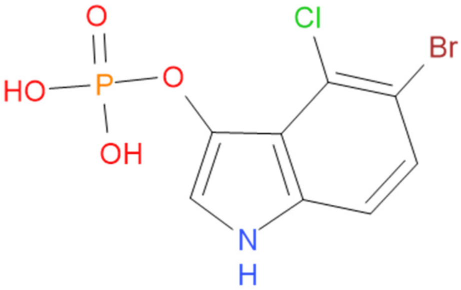

(5)

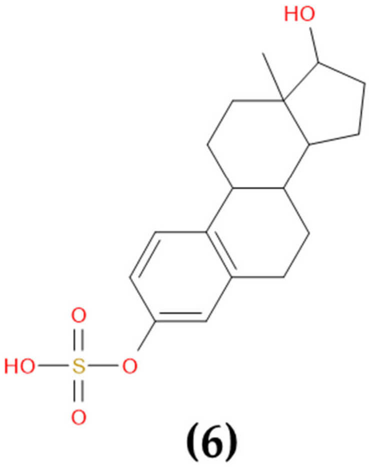

(6)

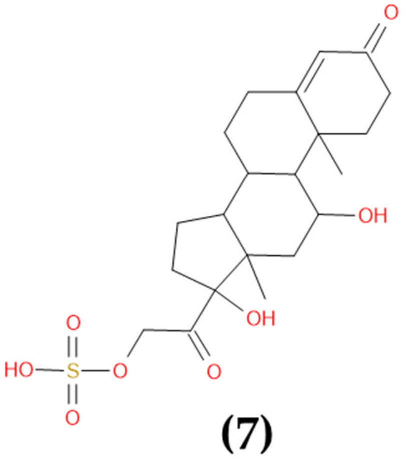

(7)

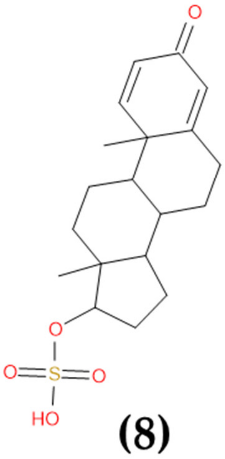

(8)

Supplementary Figure S2. Chemical structure of substrates characterized with PyuS. **(1)** – *para*-nitrocatechol sulfate (pNCS), **(2)** – 4-methylumbelliferyl sulfate (4MUS), **(3)** – *para*-nitrophenyl phosphate (pNPP), **(4)** – 5-bromo-4-chloro-3-indoxyl sulfate (X-Sulf), **(5)** – 5-bromo-4-chloro-3-indoxyl phosphate (X-Phos), **(6)** – 17α-estradiol sulfate (ES), **(7)** – cortisol 21-sulfate, and **(8)** boldenone sulfate (BS).
